# Lipoprotein(a) and incident venous thromboembolism in pre- and postmenopausal women, and in men

**DOI:** 10.1093/eurheartj/ehag252

**Published:** 2026-03-28

**Authors:** Daniel Ezzat, Diana M Lopez, Brian L Claggett, Linke Li, Niekbachsh Mohammadnia, Art Schuermans, Jan Hemeryck, Annie Chang, Samantha Murillo, Michelle L O'Donoghue, Behnood Bikdeli, Zhi Yu, Pradeep Natarajan, Aniruddh P Patel, Maria A Pabon, Michael C Honigberg

**Affiliations:** Heart and Vascular Institute, Mass General Brigham, 55 Fruit St, Boston, MA 02114, USA; Center for Genomic Medicine, Massachusetts General Hospital, 185 Cambridge Street CPZN 3.190, Boston, MA 02114, USA; Cardiovascular Disease Initiative and Program in Medical and Population Genetics, Broad Institute of MIT and Harvard, 75 Ames St, Cambridge, MA 02142, USA; Faculty of Medicine, KU Leuven, Herestraat 49, Leuven, Belgium; Heart and Vascular Institute, Mass General Brigham, 55 Fruit St, Boston, MA 02114, USA; Department of Medicine, Harvard Medical School, 25 Shattuck St, Boston, MA 02115, USA; Heart and Vascular Institute, Mass General Brigham, 55 Fruit St, Boston, MA 02114, USA; Department of Medicine, Harvard Medical School, 25 Shattuck St, Boston, MA 02115, USA; Heart and Vascular Institute, Mass General Brigham, 55 Fruit St, Boston, MA 02114, USA; Center for Genomic Medicine, Massachusetts General Hospital, 185 Cambridge Street CPZN 3.190, Boston, MA 02114, USA; Cardiovascular Disease Initiative and Program in Medical and Population Genetics, Broad Institute of MIT and Harvard, 75 Ames St, Cambridge, MA 02142, USA; Clinical and Translational Epidemiology Unit, Department of Medicine, Massachusetts General Hospital, 100 Cambridge St, Boston, MA 02114, USA; Heart and Vascular Institute, Mass General Brigham, 55 Fruit St, Boston, MA 02114, USA; Center for Genomic Medicine, Massachusetts General Hospital, 185 Cambridge Street CPZN 3.190, Boston, MA 02114, USA; Cardiovascular Disease Initiative and Program in Medical and Population Genetics, Broad Institute of MIT and Harvard, 75 Ames St, Cambridge, MA 02142, USA; Department of Cardiology, Radboud University Medical Center, Geert Grooteplein Zuid 10, Nijmegen, the Netherlands; Heart and Vascular Institute, Mass General Brigham, 55 Fruit St, Boston, MA 02114, USA; Center for Genomic Medicine, Massachusetts General Hospital, 185 Cambridge Street CPZN 3.190, Boston, MA 02114, USA; Cardiovascular Disease Initiative and Program in Medical and Population Genetics, Broad Institute of MIT and Harvard, 75 Ames St, Cambridge, MA 02142, USA; Faculty of Medicine, KU Leuven, Herestraat 49, Leuven, Belgium; Heart and Vascular Institute, Mass General Brigham, 55 Fruit St, Boston, MA 02114, USA; Center for Genomic Medicine, Massachusetts General Hospital, 185 Cambridge Street CPZN 3.190, Boston, MA 02114, USA; Cardiovascular Disease Initiative and Program in Medical and Population Genetics, Broad Institute of MIT and Harvard, 75 Ames St, Cambridge, MA 02142, USA; Faculty of Medicine, KU Leuven, Herestraat 49, Leuven, Belgium; Heart and Vascular Institute, Mass General Brigham, 55 Fruit St, Boston, MA 02114, USA; Center for Genomic Medicine, Massachusetts General Hospital, 185 Cambridge Street CPZN 3.190, Boston, MA 02114, USA; Cardiovascular Disease Initiative and Program in Medical and Population Genetics, Broad Institute of MIT and Harvard, 75 Ames St, Cambridge, MA 02142, USA; Icahn School of Medicine at Mount Sinai, 1 Gustave L. Levy Pl, New York, NY, USA; Heart and Vascular Institute, Mass General Brigham, 55 Fruit St, Boston, MA 02114, USA; Center for Genomic Medicine, Massachusetts General Hospital, 185 Cambridge Street CPZN 3.190, Boston, MA 02114, USA; Cardiovascular Disease Initiative and Program in Medical and Population Genetics, Broad Institute of MIT and Harvard, 75 Ames St, Cambridge, MA 02142, USA; Heart and Vascular Institute, Mass General Brigham, 55 Fruit St, Boston, MA 02114, USA; Department of Medicine, Harvard Medical School, 25 Shattuck St, Boston, MA 02115, USA; Heart and Vascular Institute, Mass General Brigham, 55 Fruit St, Boston, MA 02114, USA; Department of Medicine, Harvard Medical School, 25 Shattuck St, Boston, MA 02115, USA; YNHH/Yale Center for Outcomes Research and Evaluation (CORE), 195 Church St, New Haven, CT 06510, USA; Heart and Vascular Institute, Mass General Brigham, 55 Fruit St, Boston, MA 02114, USA; Center for Genomic Medicine, Massachusetts General Hospital, 185 Cambridge Street CPZN 3.190, Boston, MA 02114, USA; Cardiovascular Disease Initiative and Program in Medical and Population Genetics, Broad Institute of MIT and Harvard, 75 Ames St, Cambridge, MA 02142, USA; Clinical and Translational Epidemiology Unit, Department of Medicine, Massachusetts General Hospital, 100 Cambridge St, Boston, MA 02114, USA; Heart and Vascular Institute, Mass General Brigham, 55 Fruit St, Boston, MA 02114, USA; Center for Genomic Medicine, Massachusetts General Hospital, 185 Cambridge Street CPZN 3.190, Boston, MA 02114, USA; Cardiovascular Disease Initiative and Program in Medical and Population Genetics, Broad Institute of MIT and Harvard, 75 Ames St, Cambridge, MA 02142, USA; Department of Medicine, Harvard Medical School, 25 Shattuck St, Boston, MA 02115, USA; Heart and Vascular Institute, Mass General Brigham, 55 Fruit St, Boston, MA 02114, USA; Center for Genomic Medicine, Massachusetts General Hospital, 185 Cambridge Street CPZN 3.190, Boston, MA 02114, USA; Cardiovascular Disease Initiative and Program in Medical and Population Genetics, Broad Institute of MIT and Harvard, 75 Ames St, Cambridge, MA 02142, USA; Heart and Vascular Institute, Mass General Brigham, 55 Fruit St, Boston, MA 02114, USA; Department of Medicine, Harvard Medical School, 25 Shattuck St, Boston, MA 02115, USA; Heart and Vascular Institute, Mass General Brigham, 55 Fruit St, Boston, MA 02114, USA; Center for Genomic Medicine, Massachusetts General Hospital, 185 Cambridge Street CPZN 3.190, Boston, MA 02114, USA; Cardiovascular Disease Initiative and Program in Medical and Population Genetics, Broad Institute of MIT and Harvard, 75 Ames St, Cambridge, MA 02142, USA; Department of Medicine, Harvard Medical School, 25 Shattuck St, Boston, MA 02115, USA

**Keywords:** Lipoprotein(a), Venous thromboembolism, Menopause, Premenopause, Postmenopause, Hormone replacement therapy

## Abstract

**Background and Aims:**

Elevated lipoprotein(a) [Lp(a)] levels are an established risk factor for atherosclerotic cardiovascular disease, but the association between Lp(a) and venous thromboembolism (VTE) remains unclear. Sex and hormonal status may modify the relationship between Lp(a) and VTE.

**Methods:**

The present study included participants from the UK Biobank with available baseline Lp(a) data. Individuals with a history of VTE or cancer, as well as those using anticoagulants, were excluded. Multivariable-adjusted Cox models were used to assess the association between Lp(a) levels ≥125 nmol/L and incident VTE in premenopausal women, postmenopausal women, and men. Subgroup analyses stratified premenopausal women by oral contraceptive (OCP) use and postmenopausal women by menopausal hormone therapy (MHT) use.

**Results:**

Among 55 302 premenopausal women, 129 045 postmenopausal women, and 189 013 men, the proportions with Lp(a) ≥ 125 nmol/L were 14.0%, 19.0%, and 15.0%, respectively. Over a median (interquartile range) follow-up of 13.6 (12.9–14.4) years, 8186 VTE events occurred (cumulative incidence 2.2%). Lp(a) ≥ 125 nmol/L was associated with incident VTE in premenopausal women [adjusted hazard ratio (aHR) 1.32; 95% confidence interval (CI) 1.04–1.66; *P* = .02] but not in postmenopausal women (aHR 1.03; 95% CI 0.94–1.13; *P* = .47; *P*_interaction_ = .03) or men (aHR 1.00; 95% CI 0.92–1.08; *P* = .94). OCP use did not modify the Lp(a)-VTE association among premenopausal women (*P*_interaction_ = .61). However, among postmenopausal MHT users, Lp(a) ≥ 125 nmol/L was associated with higher VTE risk (aHR 1.48; 95% CI 1.03–2.12; *P* = .03; *P*_interaction_ = .04).

**Conclusions:**

Elevated Lp(a) was associated with VTE in premenopausal women and in postmenopausal MHT users, suggesting that hormonal context may influence Lp(a)-associated thrombotic risk.

## Structured Graphical Abstract

**Graphical Abstract:**
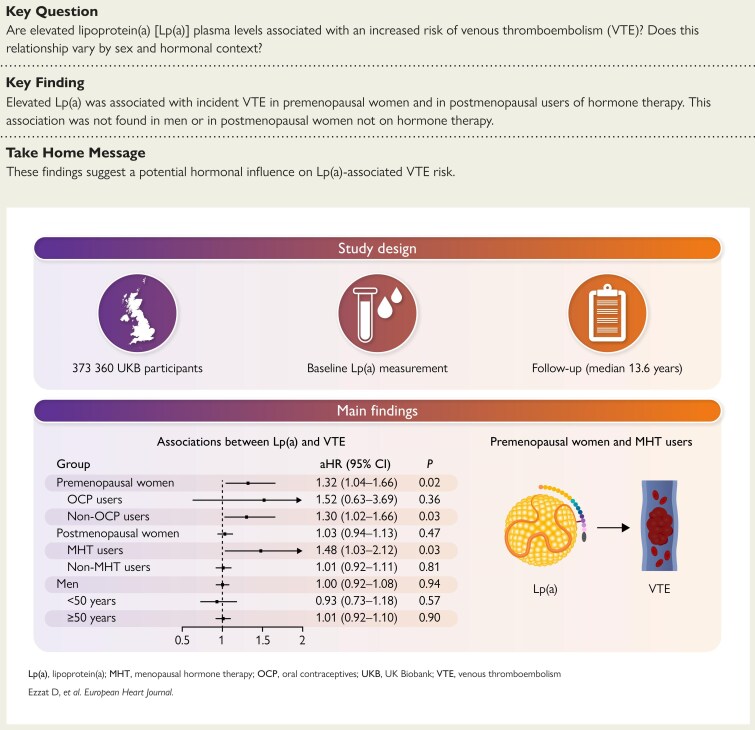

**See the editorial comment for this article ‘Two to Tango: lipoprotein(a) and hormonal status as determinants of venous thromboembolism risk', by P.R. Taub and H.S. Bhatia, https://doi.org/10.1093/eurheartj/ehag263.**

## Introduction

Lipoprotein(a) [Lp(a)] is an atherogenic lipoprotein that contains apolipoprotein B-100 covalently bound to an apolipoprotein(a) moiety, with circulating blood levels that are primarily genetically determined.^1–4^ Although preclinical studies suggest that Lp(a) may impair fibrinolysis, clinical data have yielded conflicting results.^5^ While some studies report a significant association between elevated Lp(a) levels and venous thromboembolism (VTE) risk, the overall evidence remains inconsistent and inconclusive.^6–12^

Several factors may account for these discrepancies. First, genetic studies have not demonstrated a causal relationship between Lp(a) and VTE, in contrast to the strong evidence supporting a causal role in atherosclerotic and aortic valvular heart disease.^13–15^ However, the thrombotic risk conferred by Lp(a) may be context dependent, influenced by the presence of additional thrombogenic stimuli or coexisting prothrombotic conditions.^5^ Second, heterogeneity in study design, including differences in assay standardisation, population selection, and adjustment for confounders, may also explain the inconsistent findings.

Sex and hormonal status may further modify the relationship between Lp(a) and VTE risk. Women have higher Lp(a) levels than men, and concentrations tend to increase after menopause.^3,16,17^ Moreover, exogenous oestrogen exposure via oral contraceptives (OCP) or menopausal hormone therapy (MHT) is a well-established modifiable VTE risk factor.^18–21^ These hormonal exposures may interact with elevated Lp(a) to amplify thrombotic risk. To address these knowledge gaps, we examined whether the association between Lp(a) levels and incident VTE varies by sex, menopausal status, exogenous hormone use, and oestradiol levels in 55 302 premenopausal women, 129 045 postmenopausal women, and 189 013 men in the United Kingdom (UK) Biobank.

## Methods

### Study cohort

The UK Biobank is a population-based cohort of more than 500 000 adult residents of the UK recruited between 2006 and 2010.^22^ At the baseline study visit, participants provided informed consent, completed questionnaires on health history, lifestyle, and sociodemographic factors, and underwent physical assessment and phlebotomy. The data were linked to National Health Service records, enabling identification of prevalent clinical conditions and incident events using the International Classification of Diseases (ICD), the Office of Population Censuses and Surveys classifications (OPCS), and UK death registry data. Follow-up for incident events occurred through November 2022.

**Figure 1:**
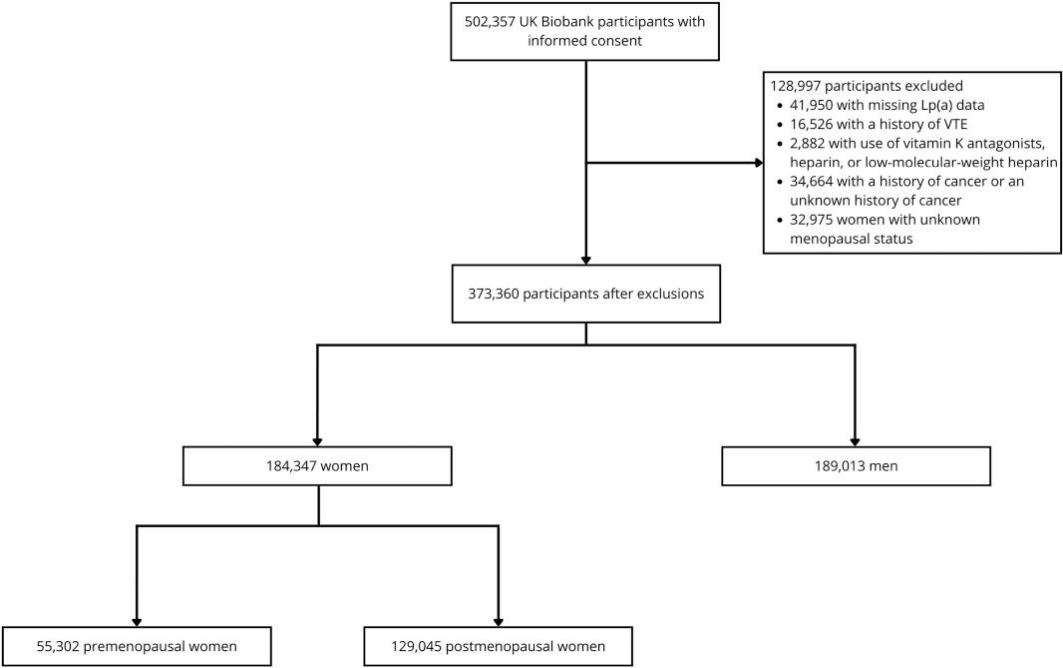
Inclusion of the study cohort. The study cohort comprised 55 302 premenopausal women, 129 045 postmenopausal women, and 189 013 men from the UK Biobank with baseline Lp(a) measurements, all of whom had no prior history of VTE or cancer and were not using anticoagulants. Lp(a), lipoprotein(a); UK, United Kingdom; VTE, venous thromboembolism

For this analysis, we included participants with available baseline Lp(a) measurements performed as a part of the research protocol. Exclusion criteria were a history of VTE, baseline use of anticoagulants, and a self-reported history of cancer or missing data on cancer history. Menopausal status was ascertained via self-report in response to the question, ‘Have you had your menopause (periods stopped)?’ Women were given five response options: ‘Yes’, ‘No’, ‘Not sure—had a hysterectomy’, ‘Not sure—other reason’, and ‘Prefer not to answer’. Only women who responded ‘Yes’ or ‘No’ were included in the present study (*Figure 1* and Supplementary data online, *Table S1*). Missing values for self-reported ethnic background (White vs non-White; *n* = 1297; 0.3%), diabetes (*n* = 870; 0.2%), and smoking status (ever vs never; *n* = 1325; 0.4%) were imputed using mode imputation. In addition, missing values for body mass index (BMI; *n* = 1366; 0.4%) were imputed using *k-nearest neighbours* (*k* = 10) via the impute.knn() function (impute package version 1.76.0 in R),^23^ incorporating age, sex, race, diabetes, smoking, cholesterol-lowering medication use, aspirin use, and Lp(a) as predictors. In total, 55 302 premenopausal women, 129 045 postmenopausal women, and 189 013 men were included. To assess whether oestrogen influences Lp(a)-associated thrombotic risk, we also examined a subgroup of 50 815 premenopausal women and 118 767 postmenopausal women with baseline oestradiol measurements.

The UK Biobank was approved by the North West Multi-centre Research Ethics Committee, and all participants provided informed consent. UK Biobank analyses were performed under application number 7089. The Mass General Brigham institutional review board approved analyses of these data.

### Lp(a) measurements

Serum Lp(a) concentrations were measured on a Beckman Coulter AU5800 using an immunoturbidimetric assay (Randox Bioscience, UK), with detectable values ranging from 3.8 to 189 nmol/L.^24–27^ Samples that fell outside this range were diluted and reanalysed, as described previously.^28^ In accordance with current guidelines, which define Lp(a) levels ≥125 nmol/L (≈50 mg/dL) as indicative of increased cardiovascular risk, Lp(a) was dichotomized into ≥125 nmol/L and <125 nmol/L for primary analyses.^1,2,29^ Additional analyses examined 105 nmol/L as an alternative threshold, as well as continuous log_2_-transformed Lp(a).

### Oestradiol measurements

Serum oestradiol concentrations were measured on a Beckman Coulter Unicel Dxl 800 using a competitive binding chemiluminescent immunoassay (Beckman Coulter, UK), with detectable values ranging from 175 to 14 588 pmol/L.^26,27^ Oestradiol values were frequently below the lower reportable range, particularly among postmenopausal women, in whom low circulating oestradiol levels are physiologically expected; therefore, values below the reportable range were considered naturally low rather than truly missing.^26^ Following oestradiol reportability in the UK Biobank guidelines (data-field 30806), we imputed half of the minimum reported value for 125 532 values below this lower limit (175 pmol/L divided by 2, i.e. 87.5 pmol/L), and the maximum reported value for 11 values above this upper limit (14 588 pmol/L). The majority of values imputed below the lower limit (111 383; 88.7%) were from postmenopausal participants.

### *LPA* genetic risk score

Sensitivity analyses examined a genetic risk score for Lp(a) in lieu of measured Lp(a). Genotypes in UK Biobank participants were generated using the UK Biobank Axiom or UK BiLEVE Axiom arrays (Affymetrix Research Service Laboratory), with imputation performed using the Haplotype Reference Consortium and UK10K + 1000 Genomes reference panels.^22^ We used a previously published *LPA* genetic risk score (GRS)^30,31^ to generate individual-level *LPA* genetic risk values among participants. The *LPA* GRS is composed of 43 single-nucleotide variants shown to be significantly associated with Lp(a) levels in independent datasets. Individual-level *LPA* GRS was calculated as the weighted sum of risk alleles across included variants, computed as the sum of the number of effect alleles at each variant multiplied by its corresponding effect size.

### Outcomes

Individuals were classified as incident VTE cases, as previously described,^32^ if they met at least one of the following criteria: (1) hospitalisation with ICD codes indicating deep vein thrombosis or pulmonary embolism; or (2) OPCS codes corresponding to surgical procedures related to VTE. All components used to define VTE are provided in Supplementary data online, *Table S2*.

### Statistical analysis

Participant characteristics were compared between individuals with Lp(a) levels ≥125 nmol/L vs <125 nmol/L. Continuous variables were compared using the Student’s *t*-test, and categorical variables were compared using the Pearson chi-squared test. To evaluate the stability of Lp(a) levels in the present cohort, we computed the mean difference between baseline and repeat Lp(a) measurements in a subset of UK Biobank participants who had repeat measurements taken in 2012–2013.

In the primary analysis, Cox proportional hazards models were used to assess the association between Lp(a) levels ≥125 nmol/L and incident VTE in premenopausal women, postmenopausal women, and men. Follow-up began at the baseline UK Biobank visit in 2006–2010, and participants who did not experience the specified event were censored at the end of follow-up. Models were adjusted for age (modelled as a linear and quadratic term), BMI, self-reported ethnic background (White vs non-White), prevalent diabetes mellitus, smoking status (ever vs never), cholesterol-lowering medication use, and aspirin use. In addition, models for premenopausal women were further adjusted for current OCP use, and models for postmenopausal women were further adjusted for current MHT use. The proportional hazards assumption was assessed using Schoenfeld residuals.

Interaction terms were tested for: (1) Lp(a) and menopausal status, (2) Lp(a) and current OCP use, and (3) Lp(a) and current MHT use. In secondary analyses, models were stratified by current OCP use in premenopausal women and current MHT use in postmenopausal women. Furthermore, subgroup analyses were performed for men aged <50 years and ≥50 years to better understand whether observations in premenopausal and postmenopausal women are attributable to sex differences or age differences. Additional age-stratified analyses in women (<50 and ≥50 years) were performed to assess age-dependent associations. In sensitivity analyses, Lp(a) was modelled as a continuous log_2_-transformed variable, with values of 0 imputed as 0.01 nmol/L (half the lowest detected positive value, 0.02 nmol/L) to enable transformation of the right-skewed Lp(a) distribution. Additional sensitivity analyses evaluated 105 nmol/L as an alternative threshold, based on a recent statement issued by the European Society of Cardiology.^33^ We further conducted sensitivity analyses under a complete-case approach, excluding individuals with missing covariate data rather than performing imputation. In addition, the use of anticoagulant therapy may influence VTE risk, but medications were systematically recorded in the full cohort only at baseline in the UK Biobank. Therefore, we conducted sensitivity analyses further excluding individuals with prevalent atrial fibrillation or flutter at baseline and additionally adjusted for incident atrial fibrillation or flutter as a time-varying covariate, to account for the primary alternative (non-VTE) indication for initiating anticoagulant therapy during follow-up. Furthermore, we performed secondary analyses differentiating between deep vein thrombosis and pulmonary embolism. Finally, we calculated population-attributable fractions $\left(PAF=\;\frac{\;prevalence\;of\;risk\;factor\;\cdot \;(aHR\;-\;1)}{\;prevalence\;of\;risk\;factor\;\cdot \;\;(aHR\;-\;1)\;+\;1}\right)$ for elevated Lp(a), obesity (BMI ≥30 kg/m^2^), current smoking, and OCP use, and evaluated interactions between Lp(a) and these same factors among premenopausal women.

In additional sensitivity analyses, we examined the association between the *LPA* GRS and incident VTE using Cox proportional hazards models adjusted for age, age^2^, genotyping array, and the first 10 principal components of genetic ancestry. The *LPA* GRS was Z-standardised and analysed in three ways: comparing the top quartile to the remaining participants, comparing the top quartile to the bottom quartile, and as a continuous variable.

In exploratory analyses, we examined whether circulating oestradiol levels modified the association between Lp(a) and incident VTE. We performed interaction testing between Lp(a) and continuous log_2_-transformed oestradiol in premenopausal women and postmenopausal women. Given the natural fluctuations of oestradiol across the menstrual cycle, we examined premenopausal women across oestradiol strata of <400, 400–<800, and ≥800 pmol/L, corresponding approximately to early follicular, mid-cycle, and luteal-phase ranges.^34,35^ Among postmenopausal women, whose oestradiol levels typically fell below the assay’s lower detection limit of 175 pmol/L (imputed as 87.5 pmol/L), we compared women with oestradiol levels below vs above this threshold.^26,34,35^ Oestradiol sensitivity analyses excluded premenopausal OCP users and postmenopausal MHT users.

Two-sided *P* < .05 was considered statistically significant for the primary analysis. Findings from secondary analyses should be considered supportive and hypothesis-generating, given the possibility of type I error. All analyses were performed in R version 4.4.3.

## Results

### Participant characteristics

Among 55 302 premenopausal women (mean [standard deviation, SD] age 46.2 [4.3] years, 4930 [8.9%] OCP users), 129 045 postmenopausal women (mean [SD] age 60.1 [5.4] years, 9289 [7.2%] MHT users), and 189 013 men (mean [SD] age 56.3 [8.2] years), median (interquartile range [IQR]) Lp(a) levels were 17.6 (7.2–69.4) nmol/L, 23.9 (8.9–85.3) nmol/L, and 17.4 (7.0–69.0) nmol/L, respectively (see Supplementary data online, *Figure S1*). Across these groups, the proportions with Lp(a) ≥ 125 nmol/L were 14.0% (*n* = 7751), 19.0% (*n* = 24 476) and 15.0% (*n* = 28 303), respectively. Among 11 681 UK Biobank participants with repeat measurements in 2012–2013 (on average 4.33 years after baseline), repeat and baseline Lp(a) concentrations were highly correlated (*r* = 0.96). Repeat Lp(a) measurements were, on average, 2.22 (95% confidence interval [CI], 1.97–2.47) nmol/L higher than baseline measurements, indicating stability of Lp(a) concentrations over time. Overall, 94.5% of total participants identified as White (*Table 1*).

**Table 1:** Baseline characteristics

	Premenopausal women (*n* = 55 302)			Postmenopausal women (*n* = 129 045)			Men (*n* = 189 013)		
	Lp(a) ≥ 125 nmol/L	Lp(a) < 125 nmol/L	*P*-value	Lp(a) ≥ 125 nmol/L	Lp(a) < 125 nmol/L	*P*-value	Lp(a) ≥ 125 nmol/L	Lp(a) < 125 nmol/L	*P*-value
Age (years)	46.5 (4.5)	46.2 (4.2)	<.001	60.2 (5.4)	60.1 (5.4)	<.001	56.6 (8.1)	56.3 (8.2)	<.001
Ethnic background (White vs non-White)	7016 (90.5)	43 720 (91.9)	<.001	23 305 (95.2)	100 103 (95.7)	<.001	26 821 (94.8)	151 697 (94.4)	.01
Smoking (ever vs never)	2772 (35.8)	16 610 (34.9)	.16	10 161 (41.5)	43 175 (41.3)	.52	14 558 (51.4)	80 150 (49.9)	<.001
Diabetes mellitus	189 (2.4)	983 (2.1)	.04	994 (4.1)	3965 (3.8)	.05	1830 (6.5)	10 799 (6.7)	.12
Body mass index (kg/m^2^)	27.2 (5.4)	26.2 (5.2)	<.001	27.2 (5.0)	27.0 (5.0)	<.001	27.6 (4.1)	27.8 (4.2)	<.001
Waist-to-hip ratio	0.808 (0.068)	0.799 (0.067)	<.001	0.823 (0.069)	0.821 (0.070)	<.001	0.933 (0.063)	0.935 (0.065)	<.001
Systolic blood pressure (mmHg)	129.3 (17.2)	127.5 (17.2)	<.001	141.4 (20.2)	140.3 (20.2)	<.001	142.8 (18.6)	142.6 (18.4)	.06
Diastolic blood pressure (mmHg)	80.1 (10.7)	78.8 (10.6)	<.001	81.4 (10.5)	81.0 (10.4)	<.001	83.9 (10.5)	84.1 (10.5)	.02
Total cholesterol (mg/dL)	220.8 (37.6)	206.5 (36.3)	<.001	241.8 (45.1)	232.7 (42.9)	<.001	218.9 (45.2)	211.5 (43.1)	<.001
HDL cholesterol (mg/dL)	60.0 (13.6)	59.5 (13.5)	.004	63.5 (14.6)	62.7 (14.8)	<.001	50.8 (11.9)	49.3 (12.0)	<.001
LDL cholesterol (mg/dL)	137.3 (29.4)	125.8 (28.5)	<.001	151.2 (34.8)	143.7 (33.3)	<.001	140.0 (34.4)	134.1 (32.9)	<.001
eGFR (mL/min/1.73 m^2^)	101.3 (12.1)	102.1 (11.6)	<.001	91.7 (12.7)	92.2 (12.3)	<.001	94.6 (12.9)	95.2 (12.8)	<.001
Aspirin use	366 (4.7)	1731 (3.6)	<.001	3156 (12.9)	11 504 (11.0)	<.001	6343 (22.4)	28 162 (17.5)	<.001
Cholesterol-lowering medication use	370 (4.8)	1092 (2.3)	<.001	4927 (20.1)	14 738 (14.1)	<.001	8344 (29.5)	32 948 (20.5)	<.001
Statins	354 (4.6)	1021 (2.1)	<.001	4589 (18.7)	13 539 (12.9)	<.001	7947 (28.1)	31 146 (19.4)	<.001
Ezetimibe	23 (0.3)	44 (0.09)	<.001	300 (1.2)	648 (0.6)	<.001	414 (1.5)	1181 (0.7)	<.001
Fibrates	2 (0.03)	16 (0.03)	.99	47 (0.2)	204 (0.2)	.99	88 (0.3)	638 (0.4)	.04
Bile acid sequestrants	2 (0.03)	13 (0.03)	1.00	8 (0.03)	43 (0.04)	.68	10 (0.04)	45 (0.03)	.63
Antihypertensive medication use	576 (7.4)	2477 (5.2)	<.001	5589 (22.8)	20 913 (20.0)	<.001	7477 (26.4)	36 891 (23.0)	<.001

Continuous variables are reported as means (standard deviations) and were compared using the independent samples *t*-test. Categorical variables are reported as *n* (%) and were compared using the Pearson chi-squared test.

eGFR, estimated glomerular filtration rate; HDL, high-density lipoprotein; LDL, low-density lipoprotein; Lp(a), lipoprotein(a).

Participants with Lp(a) concentrations <125 nmol/L served as the reference group for all sex and menopausal strata (*Table 1*). Across groups, individuals with Lp(a) concentrations ≥125 nmol/L tended to be older; had higher levels of total cholesterol, high-density lipoprotein cholesterol, and low-density lipoprotein cholesterol; had lower estimated glomerular filtration rate; and were more likely to use aspirin, cholesterol-lowering therapy, and antihypertensive medications. Among premenopausal women, those with Lp(a) ≥ 125 nmol/L were more likely to report prevalent diabetes mellitus. Both premenopausal and postmenopausal women with elevated Lp(a) had higher BMI, waist-to-hip ratio, systolic blood pressure, and diastolic blood pressure compared to those with Lp(a) < 125 nmol/L. Men with Lp(a) ≥ 125 nmol/L were more likely to report ever smoking, but had slightly lower BMI, waist-to-hip ratio, and diastolic blood pressure relative to men with Lp(a) < 125 nmol/L (*Table 1*).

### Incidence of VTE across sex and hormonal groups

**Figure 2:**
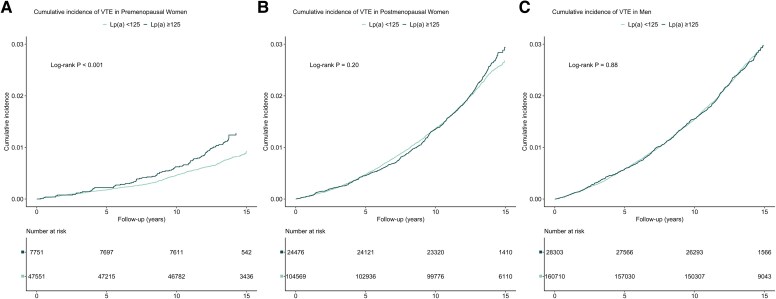
Cumulative incidence of VTE in (*A*) premenopausal women, (*B*) postmenopausal women, and (*C*) men. Cumulative incidence of VTE during follow-up, stratified by Lp(a) ≥ 125 nmol/L vs <125 nmol/L in (*A*) premenopausal women, (*B*) postmenopausal women, and (*C*) men. CI, confidence interval; HR, hazard ratio; Lp(a), lipoprotein(a); VTE, venous thromboembolism

Over a median (IQR) follow-up of 13.6 (12.9–14.4) years, incident VTE occurred in 453 (0.8%) premenopausal women, 2991 (2.3%) postmenopausal women, and 4742 (2.5%) men (*Figure 2*). Among premenopausal women, those with Lp(a) ≥ 125 nmol/L had a higher crude incidence rate of VTE per 1000 person-years (0.84; 95% CI 0.69–1.04) compared with those with Lp(a) < 125 nmol/L (0.56; 95% CI 0.51–0.62). This pattern was consistent regardless of current OCP use (see Supplementary data online, *Figure S2* and Supplementary data online, *Table S3*). In contrast, crude VTE incidence rates were similar across Lp(a) categories in both postmenopausal women (1.82; 95% CI 1.68–1.98 in Lp(a) ≥ 125 nmol/L compared to 1.72; 95% CI 1.65–1.79 in Lp(a) < 125 nmol/L) and in men (1.89; 95% CI 1.76–2.04 in Lp(a) ≥ 125 nmol/L compared to 1.91; 95% CI 1.85–1.97 in Lp(a) < 125 nmol/L). However, among postmenopausal women using MHT at baseline, those with elevated Lp(a) had a notably higher crude VTE incidence rate per 1000 person-years (2.07; 95% CI 1.51–2.85), compared with non-MHT users (1.35; 95% CI 1.15–1.59) (see Supplementary data online, *Figure S3* and Supplementary data online, *Table S3*). Supplementary data online, *Figure S4* presents cubic splines depicting the relationships of Lp(a) and the *LPA* GRS with incident VTE, with positive associations seen uniquely among premenopausal women. Supplementary data online, *Figure S5* presents the cumulative incidence of VTE in men stratified by age. Supplementary data online, *Figure S6* illustrates the cumulative incidence of VTE with age as the underlying time scale.

### Multivariable-adjusted associations between Lp(a) and incident VTE across sex and hormonal groups

There was evidence of effect modification by menopausal status in the association between Lp(a) and incident VTE in women (*P*_interaction_ = .03) (see Supplementary data online, *Table S4*). In premenopausal women, Lp(a) ≥ 125 nmol/L was associated with an increased risk of VTE (adjusted hazard ratio [aHR] 1.32; 95% CI 1.04–1.66; *P* = .02). In contrast, no significant association was observed among postmenopausal women (aHR 1.03; 95% CI 0.94–1.13; *P* = .47) or men (aHR 1.00; 95% CI 0.92–1.08; *P* = .94) (*Figure 2*). To evaluate whether this pattern was attributable to age, we conducted subgroup analyses in men younger than 50 years and those aged 50 years or older, which revealed no difference in VTE risk by Lp(a) level across age groups (*Table 2*). In age-stratified analyses among women, Lp(a) ≥ 125 nmol/L was associated with an increased risk of VTE in women under 50 years of age (aHR 1.38; 95% CI 1.06–1.80; *P* = .02), but not in women aged 50 years or older (aHR 1.04; 95% CI 0.95–1.13; *P* = .42). Of note, 44 049 (91.8%) of women aged <50 years were premenopausal and 125 088 (91.7%) of women aged ≥50 years were postmenopausal, reflecting a high correlation of menopausal status with age at baseline (see Supplementary data online, *Table S5*).

**Table 2:** Associations of Lp(a) ≥ 125 nmol/L with incident venous thromboembolism in premenopausal women, postmenopausal women, and men

	Unadjusted models		Adjusted models	
	HR (95% CI)	*P*-value	HR (95% CI)	*P*-value
Premenopausal women	1.50 (1.19–1.90)	<.001	1.32 (1.04–1.66)	.02
Postmenopausal women	1.06 (0.97–1.16)	.20	1.03 (0.94–1.13)	.47
Men	0.99 (0.92–1.08)	.88	1.00 (0.92–1.08)	.94

Cox proportional hazards models in which participants with Lp(a) < 125 nmol/L constitute the reference group. The adjusted models accounted for age, age^2^, body mass index, ethnic background, diabetes mellitus, smoking status, cholesterol-lowering medication use, and aspirin use. In addition, models for premenopausal women were further adjusted for OCP use, and models for postmenopausal women were further adjusted for MHT use.

CI, confidence interval; HR, hazard ratio; Lp(a), lipoprotein(a); MHT, menopausal hormone therapy; OCP, oral contraceptive.

Among premenopausal women, we did not observe significant effect modification by OCP use (*P*_interaction_ = .61). However, MHT use modified the association between Lp(a) and VTE among postmenopausal women (*P*_interaction_ = .04) (see Supplementary data online, *Table S3*). Specifically, those with Lp(a) ≥ 125 nmol/L who were using MHT at baseline demonstrated an increased risk of incident VTE (aHR 1.48; 95% CI 1.03–2.12; *P* = .03), whereas no association was observed among non-users (aHR 1.01; 95% CI 0.92–1.11; *P* = .81) (*Table 3*).

**Table 3:** Associations of Lp(a) ≥ 125 nmol/L with incident venous thromboembolism in subgroups of premenopausal women, postmenopausal women, and men

	Unadjusted models		Adjusted models	
	HR (95% CI)	*P*-value	HR (95% CI)	*P*-value
Premenopausal women				
OCP users (*n* = 4930)	1.75 (0.73–4.22)	.21	1.52 (0.63–3.69)	.36
Non-OCP users (*n* = 50 372)	1.48 (1.17–1.88)	.001	1.30 (1.02–1.66)	.03
Postmenopausal women				
MHT users (*n* = 9289)	1.53 (1.07–2.19)	.02	1.48 (1.03–2.12)	.03
Non-MHT users (*n* = 119 756)	1.03 (0.94–1.14)	.48	1.01 (0.92–1.11)	.81
Men				
<50 years (*n* = 46 610)	0.94 (0.74–1.19)	.61	0.93 (0.73–1.18)	.57
≥50 years (*n* = 142 403)	0.99 (0.91–1.08)	.80	1.01 (0.92–1.10)	.90

Cox proportional hazards models in which participants with Lp(a) < 125 nmol/L constitute the reference group. The adjusted models accounted for age, age^2^, body mass index, ethnic background, diabetes mellitus, smoking status, cholesterol-lowering medication use, and aspirin use.

CI, confidence interval; HR, hazard ratio; Lp(a), lipoprotein(a); MHT, menopausal hormone therapy; OCP, oral contraceptive.

Results were broadly consistent when: (1) Lp(a) was modelled as a continuous log_2_-transformed variable; (2) an Lp(a) threshold of 105 nmol/L was used; (3) a complete-case approach was applied; and (4) accounting for comorbidities that commonly warrant initiation of anticoagulant medications (see Supplementary data online, *Tables S6*–9). In addition, analyses differentiating between deep vein thrombosis and pulmonary embolism showed directionally consistent results for both VTE subtypes, albeit with reduced statistical power (see Supplementary data online, *Table S10*).

Among premenopausal women, there was no significant interaction between Lp(a) and BMI (*P*_interaction_ = .80) or current smoking (*P*_interaction_ = .47). The PAF of VTE for Lp(a) ≥ 125 nmol/L, obesity, current smoking, and OCP use was 4.6%, 19.6%, 5.5%, and 0.5%, respectively, among premenopausal women.

### Genetically predicted Lp(a) and VTE risk

As an additional sensitivity analysis, we examined genetically predicted Lp(a) using an *LPA* GRS in lieu of measured Lp(a). Consistent with our primary analyses, the top quartile of the *LPA* GRS was significantly associated with higher risk of VTE in premenopausal women, both relative to the remaining 75% of participants (aHR 1.23; 95% CI 1.00–1.52; *P* = .045) and to the bottom quartile (aHR 1.44; 95% CI 1.09–1.89; *P* = .009). Similarly, in postmenopausal women using MHT, the top quartile of the *LPA* GRS was significantly associated with incident VTE compared with both the remaining 75% of participants (aHR 1.40; 95% CI 1.03–1.92; *P* = .03) and the bottom quartile (aHR 2.05; 95% CI 1.31–3.20; *P* = .002) (see Supplementary data online, *Table S11*).

### Multivariable-adjusted associations between Lp(a) and incident VTE across oestradiol levels

Oestradiol levels significantly modified the association between Lp(a) and VTE in both premenopausal women and postmenopausal women. Each doubling of circulating oestradiol strengthened the Lp(a)-VTE association by approximately 46% (i.e. aHR_interaction_ 1.46; *P*_interaction_ < .001) in premenopausal women and 32% in postmenopausal women (*P*_interaction_ = .002) (see Supplementary data online, *Table S12*). Among premenopausal women, Lp(a) ≥ 125 nmol/L was associated with an increased risk of incident VTE in those with higher oestradiol levels. While Lp(a) ≥ 125 nmol/L was not significantly associated with VTE in premenopausal women with oestradiol <400 pmol/L (aHR 0.97; 95% CI 0.71–1.34; *P* = .87), a progressively stronger and significant association was observed at oestradiol 400–<800 pmol/L (aHR 1.82; 95% CI 1.13–2.93; *P* = .01) and oestradiol ≥800 pmol/L (aHR 3.16; 95% CI 1.57–6.39; *P* = .001). A similar pattern was observed in postmenopausal women: Lp(a) ≥ 125 nmol/L was associated with higher VTE risk in those with oestradiol levels ≥175 pmol/L (aHR 1.60; 95% CI 1.13–2.28; *P* = .009), whereas no association was observed in those with oestradiol levels <175 pmol/L (aHR 1.02; 95% CI 0.93–1.12; *P* = .70) (*Table 4*). Findings from the oestradiol analyses remained consistent after excluding premenopausal OCP users and postmenopausal MHT users (see Supplementary data online, *Tables S13*–14).

**Table 4:** Associations of Lp(a) ≥ 125 nmol/L with incident venous thromboembolism across strata of circulating oestradiol levels

	Unadjusted models		Adjusted models	
	HR (95% CI)	*P*-value	HR (95% CI)	*P*-value
Premenopausal women				
Oestradiol <400 pmol/L (*n* = 30 688)	1.12 (0.82–1.55)	.47	0.97 (0.71–1.34)	.87
Oestradiol 400-<800 pmol/L (*n* = 13 140)	2.07 (1.29–3.32)	.003	1.82 (1.13–2.93)	.01
Oestradiol ≥800 pmol/L (*n* = 6987)	3.31 (1.65–6.66)	<.001	3.16 (1.57–6.39)	.001
Postmenopausal women				
Oestradiol <175 pmol/L (*n* = 111 383)	1.05 (0.95–1.15)	.36	1.02 (0.93–1.12)	.70
Oestradiol ≥175 pmol/L (*n* = 7384)	1.68 (1.18–2.38)	.004	1.60 (1.13–2.28)	.009

Cox proportional hazards models in which participants with Lp(a) < 125 nmol/L constitute the reference group. The adjusted models accounted for age, age^2^, body mass index, ethnic background, diabetes mellitus, smoking status, cholesterol-lowering medication use, and aspirin use. In addition, models for premenopausal women were further adjusted for OCP use, and models for postmenopausal women were further adjusted for MHT use.

CI, confidence interval; HR, hazard ratio; Lp(a), lipoprotein(a); MHT, menopausal hormone therapy; OCP, oral contraceptive; VTE, venous thromboembolism.

## Discussion

In this large population-based cohort of 373 360 participants, elevated Lp(a) was associated with a higher risk of incident VTE in early midlife premenopausal women, but not in postmenopausal women or in men. Among postmenopausal women, subgroup analyses suggested an association between elevated Lp(a) and higher VTE risk in those using MHT, whereas no clear evidence of effect modification was observed with OCP use in premenopausal women. Furthermore, exploratory analyses suggested that higher circulating oestradiol levels may strengthen the Lp(a)-VTE association, while those with lower oestradiol levels showed no evidence of excess risk. Together, these findings suggest that the association between elevated Lp(a) and VTE risk may depend on the hormonal milieu at the time of Lp(a) measurement, with a stronger association observed in oestrogen-rich environments.

Lp(a) has long been hypothesized to influence VTE risk primarily by impairing fibrinolysis, the process by which plasmin enzymatically degrades fibrin clots to restore normal blood flow.^5,36^ The apolipoprotein(a) component of Lp(a) shares significant structural homology with plasminogen, the precursor of plasmin.^37,38^ Because of this structural similarity, Lp(a) can compete with plasminogen for binding sites on fibrin and endothelial cells, reducing plasmin formation and thereby slowing clot breakdown.^39–46^ Beyond competitive inhibition, Lp(a) has also been shown to attenuate both tissue plasminogen activator (tPA)-dependent and tPA-independent plasminogen activation, increase endothelial expression of plasminogen activator inhibitor-1, and reduce fibrin clot permeability, potentially favouring the persistence of fibrin-rich thrombi.^47–50^ Additional prothrombotic properties of Lp(a) have also been proposed, including increased tissue factor expression, inhibition of tissue factor pathway inhibitor, and promoting platelet activation and aggregation.^51–58^ Collectively, these mechanisms support the biological plausibility of an association between elevated Lp(a) and VTE.

Despite this biologic plausibility demonstrated *in vitro* and in animal models, clinical data linking Lp(a) to VTE are inconsistent.^5–12^ Retrospective case-control studies and meta-analyses have generally reported a modest association, particularly at elevated Lp(a) concentrations (>30 mg/dL, or 75 nmol/L).^6–9^ In contrast, most prospective studies have not identified an association between Lp(a) and incident VTE.^10–12^ Similarly, Mendelian randomisation studies have shown no significant increase in VTE risk with genetically elevated Lp(a) levels among pooled midlife adults.^13^ These discrepancies likely reflect substantial heterogeneity in study design, including variability in Lp(a) assays, population selection (with limited representation of premenopausal women), adjustment for thrombophilic risk factors, and the limited characterisation of sex-specific or hormone-related influences. Finally, to our knowledge, prior studies have not systematically evaluated hormonal context as an effect modifier of the Lp(a)-incident VTE association. Our study directly addresses this gap by integrating menopausal status, exogenous hormone use, and circulating oestradiol. Moreover, sensitivity analyses using the *LPA* GRS showed associations that were broadly concordant with analyses using measured Lp(a), providing supportive evidence for an association of Lp(a) with increased VTE risk within specific hormonal contexts.

The association of Lp(a) levels with thrombotic risk may be context-dependent.^5,12,59^ In our cohort of premenopausal women, who have markedly higher endogenous oestradiol than postmenopausal women,^60–64^ elevated Lp(a) was associated with a higher risk of VTE, whereas no association was observed in postmenopausal women or in men. In contrast, in secondary analyses, elevated Lp(a) appeared to be associated with increased VTE risk only in postmenopausal women using MHT, a well-established modifiable risk factor for VTE.^18,19^ Importantly, our exploratory oestradiol analyses further support a possible hormone-dependent pattern, suggesting that higher circulating oestradiol levels may strengthen the association between elevated Lp(a) and VTE in both premenopausal and postmenopausal women. Use of exogenous hormone therapy is associated with lower circulating Lp(a) levels,^65^ and the menopause transition is associated with an increase in Lp(a).^3,16,17^ The presence of elevated Lp(a) despite a high-oestrogen state may mark a particularly high-risk subgroup, as shown previously for Lp(a)-associated risk of coronary heart disease.^65^ However, prospective evidence demonstrating that Lp(a)-guided hormone selection or dosing, or thromboprophylaxis strategies, improves clinical outcomes is needed to modify broader clinical practice.

### Limitations

Our findings should be interpreted in the context of limitations. First, while this study benefits from a large sample size, the available UK Biobank data lack granularity regarding OCP and MHT use, including formulation (e.g. oestrogen-only vs combined MHT), route of administration (e.g. oral vs transdermal MHT), dose, and duration, all of which influence VTE risk. In addition, not all factors that may affect VTE risk (e.g. recent immobilisation, family history of VTE) were available in the UK Biobank. Second, given that hormonal status and exposures evolve over time, our findings should be interpreted as reflecting baseline risk stratification rather than time-updated causal effects. Menopausal status and exogenous hormone use were only systematically available at baseline. Therefore, potential transitions to postmenopause or changes in hormone therapy during follow-up were not uniformly captured, precluding sensitivity analyses accounting for dynamic changes in hormonal status. Future studies may benefit from systematic longitudinal data collection to further refine risk estimation. Third, VTE events were identified through linked administrative and hospital records without central adjudication, introducing the potential for outcome misclassification. However, these ICD and OPCS codes have recapitulated known epidemiologic and genetic associations in previous studies.^32,66^ Fourth, although we used the ≥125 nmol/L threshold recommended by the American Heart Association and others,^1,2,29^ it is unknown whether this cutoff is optimal for VTE risk; however, results were consistent when using the ≥105 nmol/L threshold proposed in the recent European Society of Cardiology statement^33^ and when modelling Lp(a) as a continuous variable. Fifth, circulating oestradiol was measured using an immunoassay with many values below the limit of detection, particularly in postmenopausal women, and at a single, non-standardised time point. Estimates should therefore be interpreted with caution, as imputation and categorisation into physiological strata may have influenced their precision. Sixth, secondary and exploratory analyses were not corrected for multiple hypothesis testing and should be considered hypothesis-generating. Finally, the UK Biobank included only women aged 40 years or older at baseline, and most participants identified as White. In addition, the UK Biobank consists of volunteer participants, which may not fully reflect the general population. These factors limit the generalisability of our findings to younger women and to more diverse populations with greater variability in both Lp(a) concentrations and VTE risk. Further validation in multiethnic cohorts with detailed reproductive and hormonal profiling will be essential.

## Conclusion

In the UK Biobank, an elevated Lp(a) level ≥125 nmol/L was associated with incident VTE only in select hormonal contexts—premenopausal women and postmenopausal women using MHT—while no association was observed in postmenopausal women not using MHT or in men. These findings may help reconcile prior inconsistent literature and support the possibility of a hormone-dependent pattern of Lp(a)-associated VTE risk. Future studies in more diverse populations and settings are needed to determine whether elevated Lp(a) should inform VTE risk assessment and counselling around exogenous oestrogen use.

## Supplementary Material

ehag252_Supplementary_Data

## Declarations

### Data Availability

UK Biobank data are available to approved investigators by application (https://www.ukbiobank.ac.uk/).
