# Supplementary material for: Lipoprotein(a) and incident venous thromboembolism in pre- and postmenopausal women, and in men
Source: Eur Heart J. 2026 Mar 28;47(27):3576–87. doi: 10.1093/eurheartj/ehag252 (PMC13266305; doi:10.1093/eurheartj/ehag252)
Supplement: ehag252_Supplementary_Data [file ehag252_supplementary_data.docx]

**Supplemental Material**

***Lipoprotein(a) and Incident Venous Thromboembolism in Premenopausal Women, Postmenopausal Women, and Men***

Daniel Ezzat, Diana M. Lopez, Brian L. Claggett, Linke Li, Niekbachsh Mohammadnia,
Art Schuermans, Jan Hemeryck, Annie Chang, Samantha Murillo, Michelle L. O'Donoghue, Behnood Bikdeli, Zhi Yu, Pradeep Natarajan, Aniruddh P. Patel, Maria A. Pabon,
Michael C. Honigberg

Supplemental Figures – page 3-8

Supplemental Tables – page 9-24

Table of Contents

[Supplemental Figures 3](#_Toc224699844)

[Supplemental Figure 1: Distribution of Lp(a) levels among (A) premenopausal women, (B) postmenopausal women, and (C) men. 3](#_Toc224699845)

[Supplemental Figure 2: Cumulative incidence of VTE in premenopausal women stratified by (A) OCP use and (B) non-OCP use. 4](#_Toc224699846)

[Supplemental Figure 3: Cumulative incidence of VTE in postmenopausal women stratified by (A) MHT use and (B) non-MHT use. 5](#_Toc224699847)

[Supplemental Figure 4: Cubic splines in premenopausal women (A and B), postmenopausal women (C and D), and men (E and F). 6](#_Toc224699848)

[Supplemental Figure 5: Cumulative incidence of VTE in men stratified by (A) age <50 years and (B) age ≥50 years. 7](#_Toc224699849)

[Supplemental Figure 6: Cumulative incidence of VTE with age as the underlying time scale in (A) premenopausal women, (B) postmenopausal women, and (C) men. 8](#_Toc224699850)

[Supplemental Tables 9](#_Toc224699851)

[Supplemental Table 1: Codes used to identify medication use. 9](#_Toc224699852)

[Supplemental Table 2: Codes used to define prevalent and incident VTE. 10](#_Toc224699853)

[Supplemental Table 3: Incidence of venous thromboembolism. 11](#_Toc224699854)

[Supplemental Table 4: Interaction terms for Lp(a) ≥125 nmol/L with menopausal status, Lp(a) ≥125 nmol/L with current OCP use in premenopausal women, and Lp(a) ≥125 nmol/L with current MHT use in postmenopausal women. 13](#_Toc224699855)

[Supplemental Table 5: Associations of Lp(a) ≥125 nmol/L with incident VTE in women stratified by age. 14](#_Toc224699856)

[Supplemental Table 6: Associations of continuous log_2_-transformed Lp(a) with incident VTE. 15](#_Toc224699857)

[Supplemental Table 7: Associations of Lp(a) ≥105 nmol/L with incident VTE. 16](#_Toc224699858)

[Supplemental Table 8: Associations of Lp(a) ≥125 nmol/L with incident VTE using a complete-case approach. 17](#_Toc224699859)

[Supplemental Table 9: Associations of Lp(a) ≥125 nmol/L with incident VTE, accounting for prevalent and incident atrial fibrillation or flutter. 18](#_Toc224699860)

[Supplemental Table 10: Associations of Lp(a) ≥125 nmol/L with incident venous thromboembolism, pulmonary embolism (with or without deep vein thrombosis), deep vein thrombosis (with or without pulmonary embolism), and isolated deep vein thrombosis. 19](#_Toc224699861)

[Supplemental Table 11: Associations of the *LPA* GRS with incident VTE. 20](#_Toc224699862)

[Supplemental Table 12: Interaction terms for Lp(a) ≥125 nmol/L with log_2_-transformed oestradiol. 22](#_Toc224699863)

[Supplemental Table 13: Interaction terms for Lp(a) ≥125 nmol/L with log_2_-transformed oestradiol, excluding OCP and MHT users. 23](#_Toc224699864)

[Supplemental Table 14: Associations of Lp(a) ≥125 nmol/L with incident VTE across strata of circulating oestradiol levels, excluding OCP and MHT users. 24](#_Toc224699865)

# Supplemental Figures

## Supplemental Figure 1: Distribution of Lp(a) levels among (A) premenopausal women, (B) postmenopausal women, and (C) men.


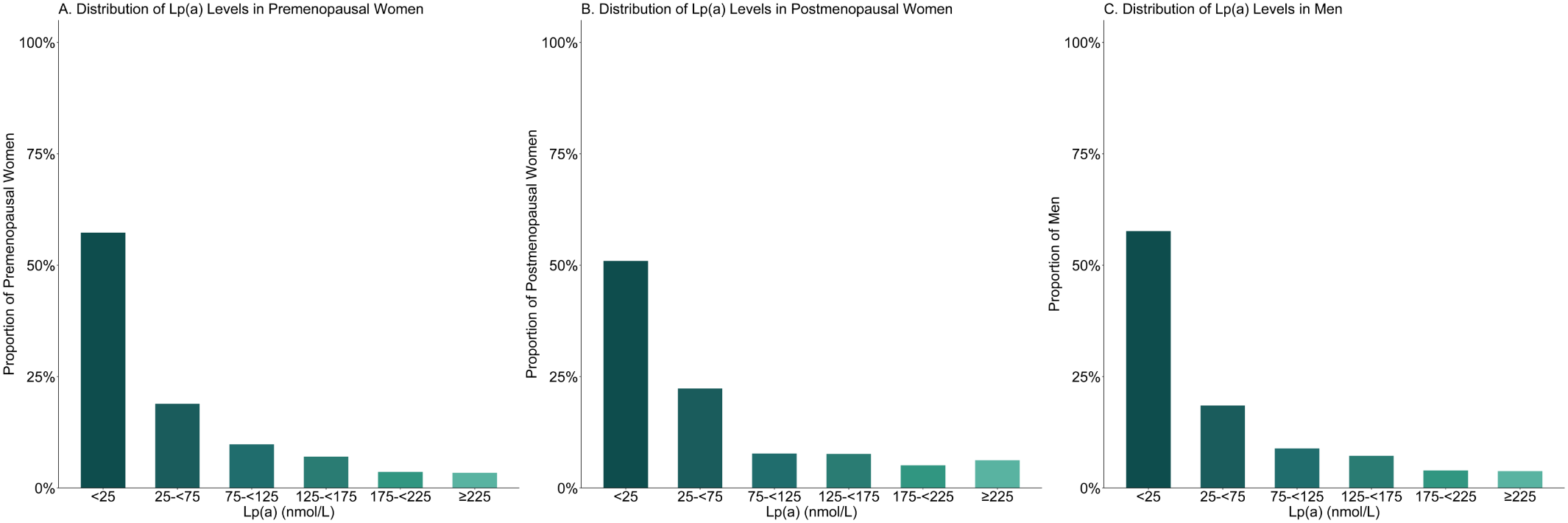


Supplemental Figure 1 shows the distribution of Lp(a) levels among (A) premenopausal women, (B) postmenopausal women, and (C) men, with each bar representing the proportion of the total population within each Lp(a) category. Lp(a), lipoprotein(a)

## Supplemental Figure 2: Cumulative incidence of VTE in premenopausal women stratified by (A) OCP use and (B) non-OCP use.


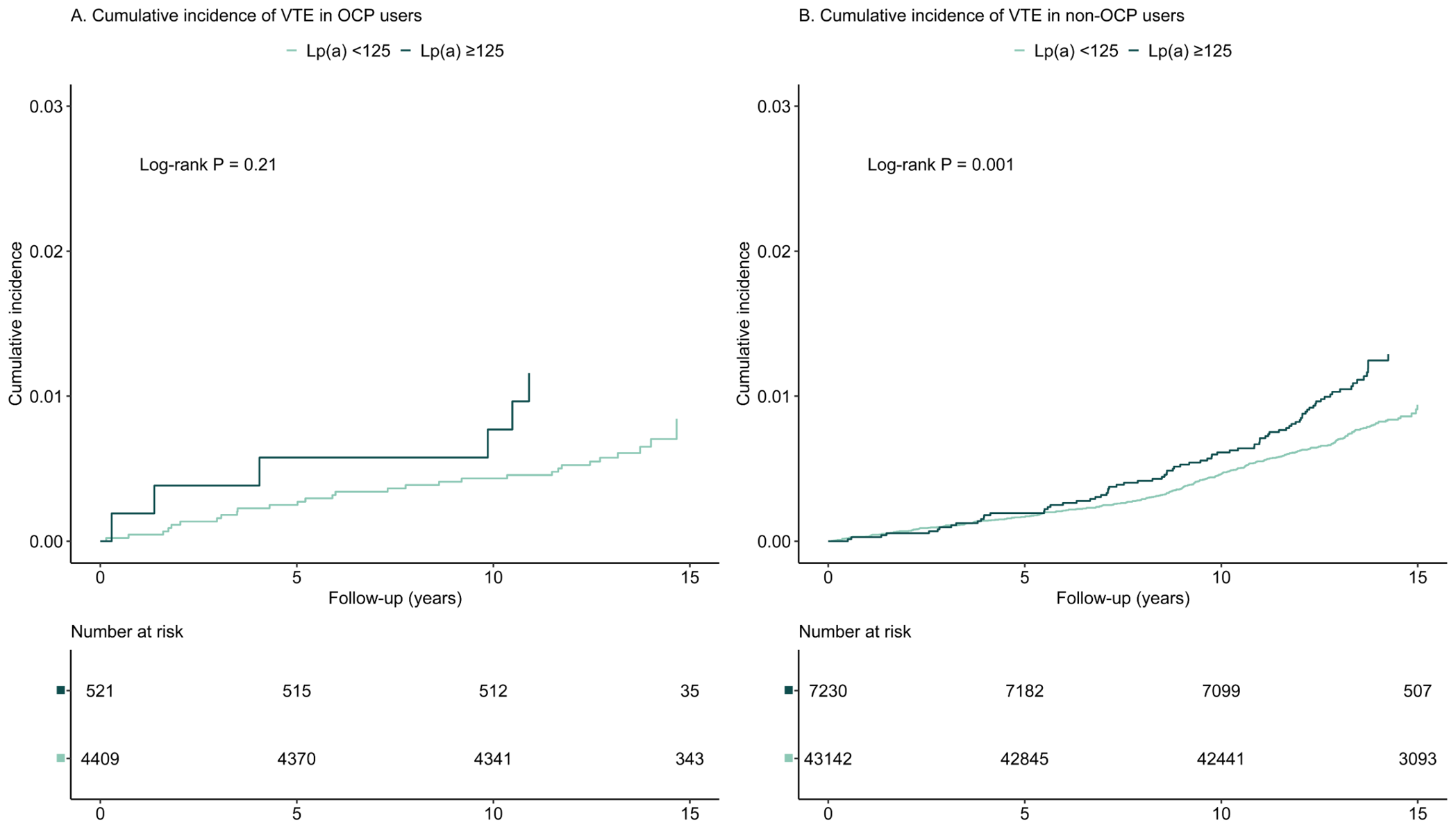


Cumulative incidence of VTE during follow-up, stratified by Lp(a) ≥125 nmol/L vs. <125 nmol/L in (A) OCP users and (B) non-OCP users. Lp(a), lipoprotein(a); OCP, oral contraceptive; VTE, venous thromboembolism

## Supplemental Figure 3: Cumulative incidence of VTE in postmenopausal women stratified by (A) MHT use and (B) non-MHT use.


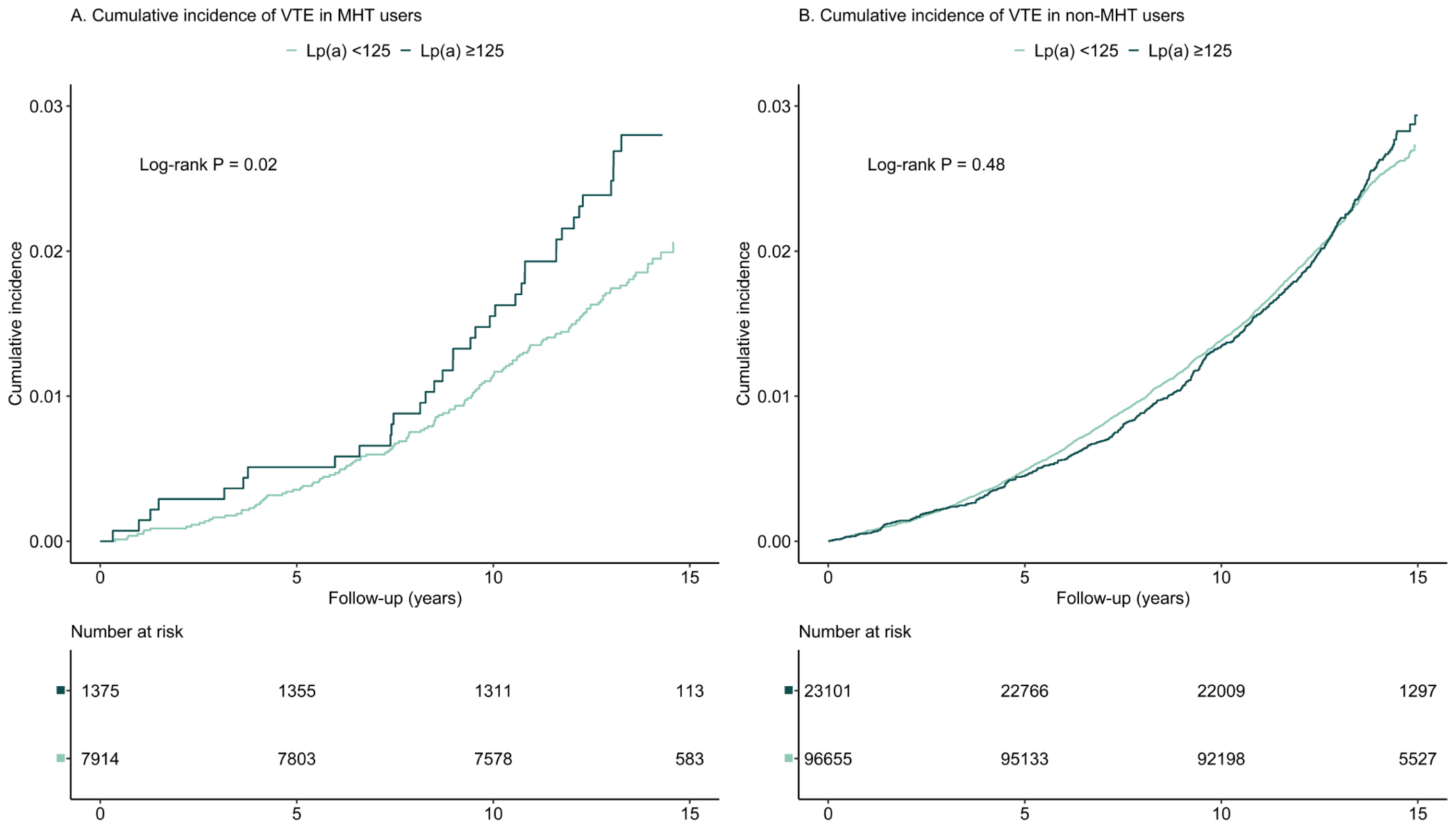


Cumulative incidence of VTE during follow-up, stratified by Lp(a) ≥125 nmol/L vs. <125 nmol/L in (A) MHT users and (B) non-MHT users. Lp(a), lipoprotein(a); MHT, menopausal hormone therapy; VTE, venous thromboembolism

## Supplemental Figure 4: Cubic splines in premenopausal women (A and B), postmenopausal women (C and D), and men (E and F).


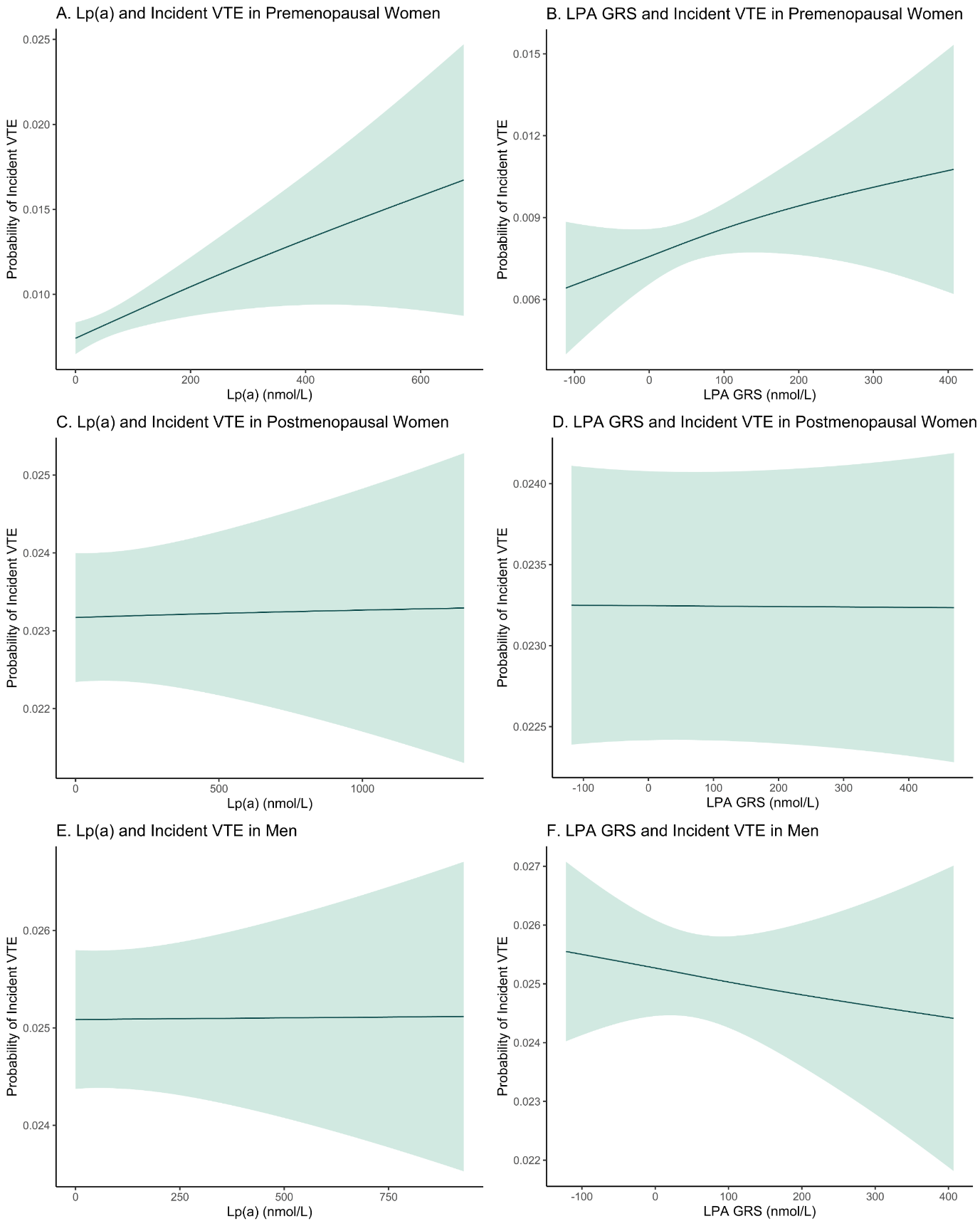


GRS, genetic risk score; Lp(a), lipoprotein(a); VTE, venous thromboembolism

## Supplemental Figure 5: Cumulative incidence of VTE in men stratified by (A) age <50 years and (B) age ≥50 years.


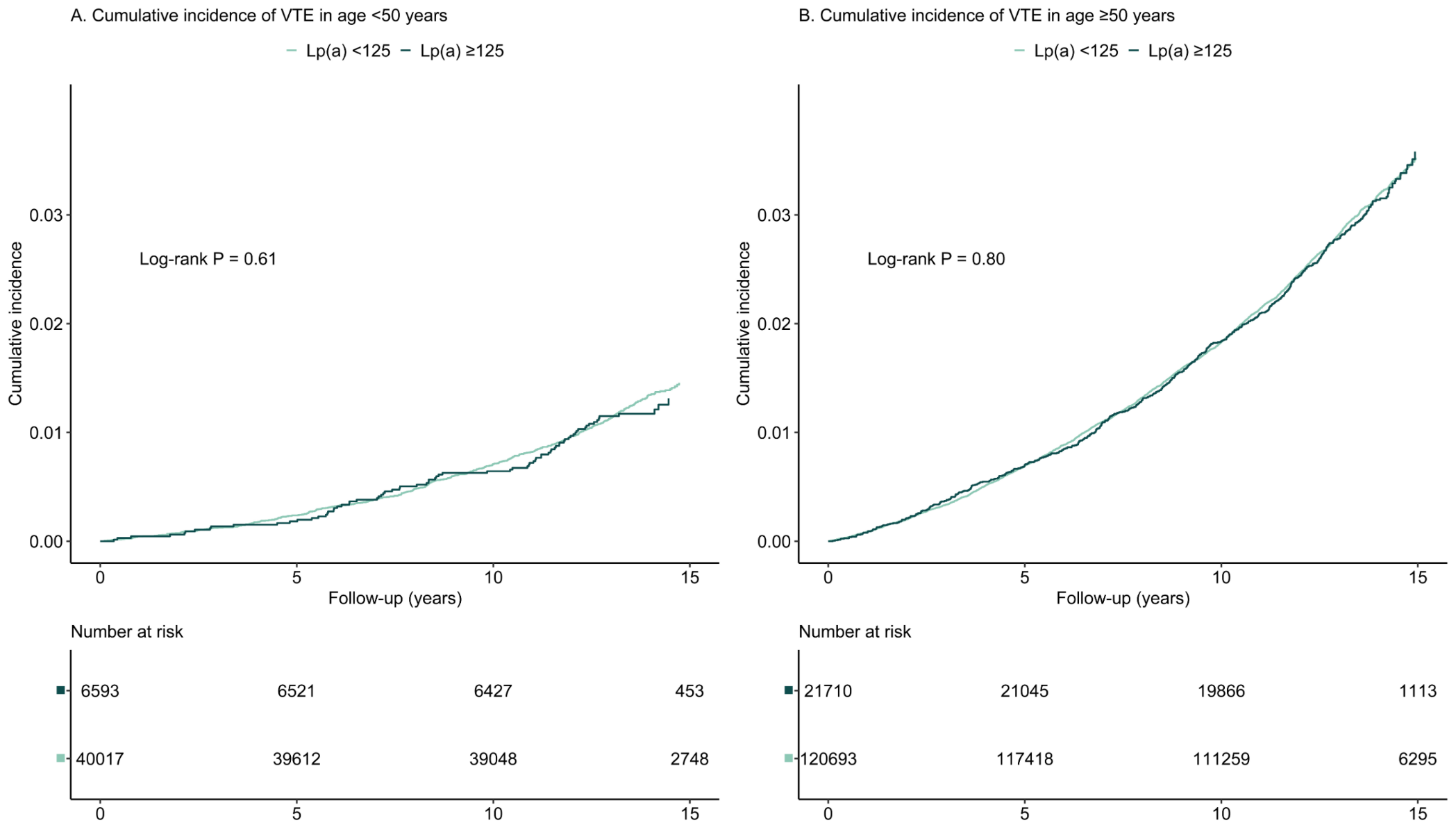


Cumulative incidence of VTE during follow-up, stratified by Lp(a) ≥125 nmol/L vs. <125 nmol/L in (A) men aged <50 years and (B) men aged ≥50 years. Lp(a), lipoprotein(a); VTE, venous thromboembolism

## Supplemental Figure 6: Cumulative incidence of VTE with age as the underlying time scale in (A) premenopausal women, (B) postmenopausal women, and (C) men.


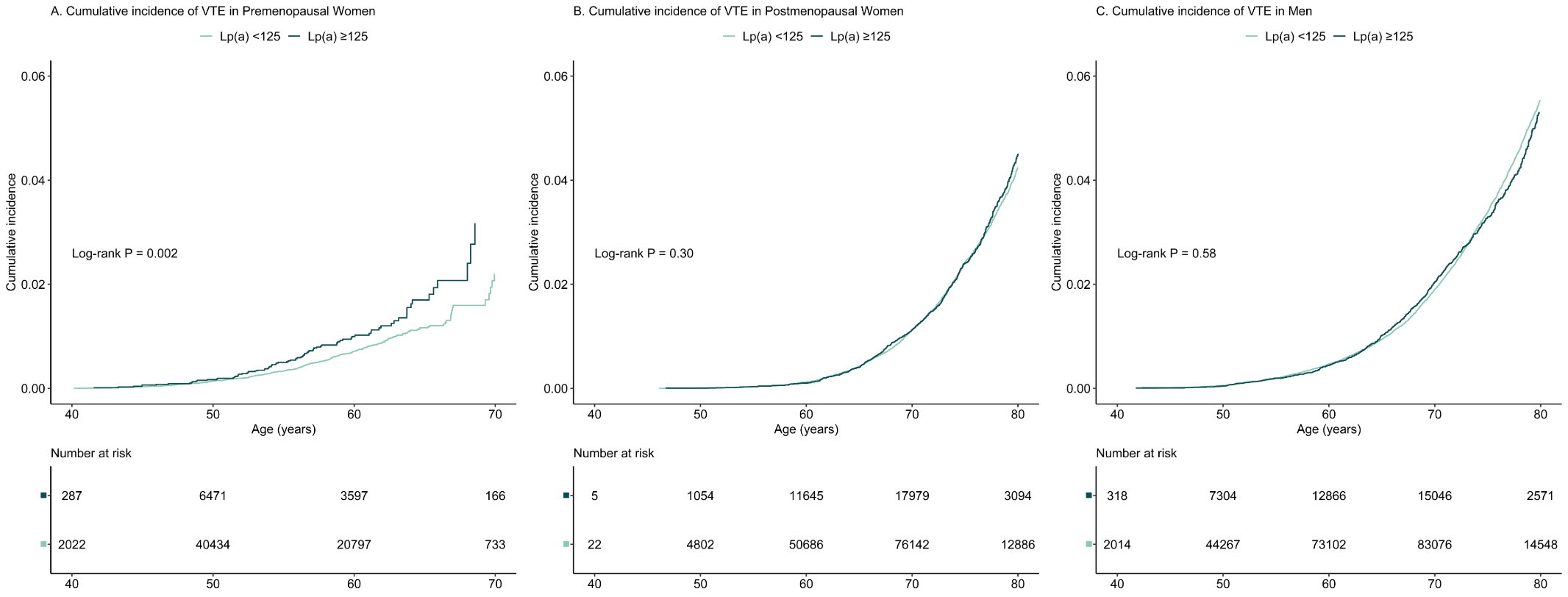


Cumulative incidence of VTE with age as the underlying time scale, stratified by Lp(a) ≥125 nmol/L vs. <125 nmol/L in (A) premenopausal women, (B) postmenopausal women, and (C) men. Lp(a), lipoprotein(a); VTE, venous thromboembolism

# Supplemental Tables

## Supplemental Table 1: Codes used to identify medication use.

| **Medication use** | **Codes in UK Biobank data field 20003** |
| --- | --- |
| Anticoagulants | 1140888266, 1140861696, 1140861698, 1140861702, 1140888204, 1140888206, 1140861506, 1140861568, 1140861574, 1140861578, 1140861584, 1140861588, 1140861594, 1140861602, 1140861604, 1140881842, 1140864956 |
| Aspirin | 1140861806, 1140864860, 1140868226, 1140868282, 1140872040, 1140882108, 1140882190, 1140882268, 1140882392, 1141163138, 1141164044, 1141167844 |
| Statins | 1140861958, 1140861970, 1140888594, 1140888648, 1140910632, 1140910654, 1141146138, 1141146234, 1141192410, 1141192414, 1140864592, 1140881748, 1141200040 |
| Fibrates | 1140861924, 1140861926, 1140861928, 1140861944, 1140861946, 1140861954, 1140862026, 1140862028, 1141157260, 1140861856, 1140861858, 1141157262 |
| Ezetimibe | 1141192736, 1141192740 |
| Bile acid sequestrants | 1140861942, 1140865576, 1141157416, 1140861936, 1140888590, 1140861848, 1140888590 |

The components used to define medication use are listed in Supplemental Table 1. All array indices (0-47) of data field 20003 were included in this definition. In addition, use of cholesterol-lowering and antihypertensive medications, oral contraceptives, and menopausal hormone therapy was defined using data fields 6153 and 6177, 6153 and 2804, and 6153 and 3546, respectively.

## Supplemental Table 2: Codes used to define prevalent and incident VTE.

| **Data field** | **Code** |
| --- | --- |
| 20002 | 1068, 1093, 1094 |
| 41202 | I26, I26.0, I26.9, I80.1, I80.2, I82.2 |
| 41204 | I26, I26.0, I26.9, I80.1, I80.2, I82.2 |
| 40001 | I26, I26.0, I26.9, I80.1, I80.2, I82.2 |
| 40002 | I26, I26.0, I26.9, I80.1, I80.2, I82.2 |
| 41200 | L79.1, L90.2 |
| 41210 | L79.1, L90.2 |
| 41203 | 4151, 4511 |
| 41205 | 4151, 4511 |
| 6152 | 5, 7 |

The components used to define VTE are listed in Supplemental Table 2. ICD-based quality control of the final cohort identified no individuals with portal vein thrombosis (I81), Budd-Chiari syndrome (I82.0), superficial or unspecified thrombophlebitis (I80.0, I80.3, I80.8, I80.9), or known coagulation disorders (D68). ICD, International Classification of Diseases; VTE, venous thromboembolism

## Supplemental Table 3: Incidence of venous thromboembolism.

|  | **Cumulative incidence, number (%)** | | **Incidence rate per 1,000 person-years of follow-up (95% CI)** | |
| --- | --- | --- | --- | --- |
|  | **Lp(a) ≥125 nmol/L** | **Lp(a) <125 nmol/L** | **Lp(a) ≥125 nmol/L** | **Lp(a) <125 nmol/L** |
| **Premenopausal women** | 89 (1.1%) | 364 (0.8%) | 0.84 (0.69-1.04) | 0.56 (0.51-0.62) |
| **OCP users** | 6 (1.2%) | 29 (0.7%) | 0.85 (0.38-1.88) | 0.48 (0.34-0.69) |
| **Non-OCP users** | 83 (1.1%) | 335 (0.8%) | 0.84 (0.68-1.05) | 0.57 (0.51-0.63) |
| **Postmenopausal women** | 594 (2.4%) | 2,397 (2.3%) | 1.82 (1.68-1.98) | 1.72 (1.65-1.79) |
| **MHT users** | 38 (2.8%) | 143 (1.8%) | 2.07 (1.51-2.85) | 1.35 (1.15-1.59) |
| **Non-MHT users** | 556 (2.4%) | 2,254 (2.3%) | 1.81 (1.66-1.96) | 1.75 (1.68-1.82) |

| **Men** | 703 (2.5%) | 4,039 (2.5%) | 1.89 (1.76-2.04) | 1.91 (1.85-1.97) |
| --- | --- | --- | --- | --- |
| **<50 years** | 78 (1.2%) | 504 (1.3%) | 0.87 (0.70-1.09) | 0.93 (0.85-1.01) |
| **≥50 years** | 625 (2.9%) | 3,535 (2.9%) | 2.22 (2.05-2.40) | 2.25 (2.17-2.32) |

CI, confidence interval; Lp(a), lipoprotein(a); VTE, venous thromboembolism

## Supplemental Table 4: Interaction terms for Lp(a) ≥125 nmol/L with menopausal status, Lp(a) ≥125 nmol/L with current OCP use in premenopausal women, and Lp(a) ≥125 nmol/L with current MHT use in postmenopausal women.

|  | **Incident VTE** | |
| --- | --- | --- |
|  | **aHR (95% CI)** | ***P_interaction_*** |
| **Interaction between Lp(a) and menopausal status** | 0.76 (0.59-0.98) | 0.03 |
| **Interaction between Lp(a) and OCP use** | 1.26 (0.51-3.15) | 0.61 |
| **Interaction between Lp(a) and MHT use** | 1.47 (1.01-2.12) | 0.04 |

Supplemental Table 4 presents Cox proportional hazards models, adjusted for age, age^2^, body mass index, ethnic background, diabetes mellitus, smoking status, cholesterol-lowering medication use, and aspirin use. aHR, adjusted hazard ratio; CI, confidence interval; Lp(a), lipoprotein(a); MHT, menopausal hormone therapy; OCP, oral contraceptive; VTE, venous thromboembolism

## Supplemental Table 5: Associations of Lp(a) ≥125 nmol/L with incident VTE in women stratified by age.

|  | **Incident VTE** | |
| --- | --- | --- |
|  | **aHR (95% CI)** | **P-value** |
| **Women <50 years**  **(n = 48,006)** | 1.38 (1.06-1.80) | 0.02 |
| **Women ≥50 years**  **(n = 136,341)** | 1.04 (0.95-1.13) | 0.42 |

Supplemental Table 5 presents Cox proportional hazards models, adjusted for age, age^2^, body mass index, ethnic background, diabetes mellitus, smoking status, cholesterol-lowering medication use, aspirin use, and exogenous hormone use. aHR, adjusted hazard ratio; CI, confidence interval; Lp(a), lipoprotein(a); VTE, venous thromboembolism

## Supplemental Table 6: Associations of continuous log_2_-transformed Lp(a) with incident VTE.

|  | **Incident VTE** | |
| --- | --- | --- |
|  | **aHR (95% CI)** | **P-value** |
| **Premenopausal women**  **(n = 55,302)** | 1.05 (1.00-1.10) | 0.05 |
| **OCP users**  **(n = 4,930)** | 1.04 (0.88-1.23) | 0.65 |
| **Non-OCP users**  **(n = 50,372)** | 1.05 (1.00-1.10) | 0.06 |
| **Postmenopausal women**  **(n = 129,045)** | 1.01 (0.99-1.02) | 0.55 |
| **MHT users**  **(n = 9,289)** | 1.07 (1.00-1.15) | 0.047 |
| **Non-MHT users**  **(n = 119,756)** | 1.00 (0.98-1.02) | 0.91 |
| **Men**  **(n = 189,013)** | 1.01 (0.99-1.02) | 0.23 |
| **<50 years**  **(n = 46,610)** | 1.02 (0.98-1.06) | 0.29 |
| **≥50 years**  **(n = 142,403)** | 1.01 (0.99-1.02) | 0.38 |

Supplemental Table 6 presents Cox proportional hazards models, adjusted for age, age^2^, body mass index, ethnic background, diabetes mellitus, smoking status, cholesterol-lowering medication use, and aspirin use. In addition, models for premenopausal women were further adjusted for OCP use, and models for postmenopausal women were further adjusted for MHT use. aHR, adjusted hazard ratio; CI, confidence interval; Lp(a), lipoprotein(a); MHT, menopausal hormone therapy; OCP, oral contraceptive; VTE, venous thromboembolism

## Supplemental Table 7: Associations of Lp(a) ≥105 nmol/L with incident VTE.

|  | **Incident VTE** | |
| --- | --- | --- |
|  | **aHR (95% CI)** | **P-value** |
| **Premenopausal women**  **(n = 55,302)** | 1.26 (1.02-1.57) | 0.04 |
| **OCP users**  **(n = 4,930)** | 1.34 (0.58-3.09) | 0.49 |
| **Non-OCP users**  **(n = 50,372)** | 1.26 (1.00-1.58) | 0.048 |
| **Postmenopausal women**  **(n = 129,045)** | 1.03 (0.94-1.12) | 0.55 |
| **MHT users**  **(n = 9,289)** | 1.42 (1.01-1.99) | 0.04 |
| **Non-MHT users**  **(n = 119,756)** | 1.01 (0.92-1.10) | 0.89 |
| **Men**  **(n = 189,013)** | 0.99 (0.92-1.07) | 0.88 |
| **<50 years**  **(n = 46,610)** | 0.98 (0.79-1.21) | 0.83 |
| **≥50 years**  **(n = 142,403)** | 1.00 (0.92-1.08) | 0.93 |

Supplemental Table 7 presents Cox proportional hazards models in which participants with Lp(a) <105 nmol/L constitute the reference group. Models were adjusted for age, age^2^, body mass index, ethnic background, diabetes mellitus, smoking status, cholesterol-lowering medication use, and aspirin use. In addition, models for premenopausal women were further adjusted for OCP use, and models for postmenopausal women were further adjusted for MHT use. aHR, adjusted hazard ratio; CI, confidence interval; Lp(a), lipoprotein(a); MHT, menopausal hormone therapy; OCP, oral contraceptive; VTE, venous thromboembolism; *Denotes statistical significance before rounding.

## Supplemental Table 8: Associations of Lp(a) ≥125 nmol/L with incident VTE using a complete-case approach.

|  | **Incident VTE** | |
| --- | --- | --- |
|  | **aHR (95% CI)** | **P-value** |
| **Premenopausal women**  **(n = 54,789)** | 1.33 (1.05-1.68) | 0.02 |
| **OCP users**  **(n = 4,900)** | 1.52 (0.63-3.70) | 0.35 |
| **Non-OCP users**  **(n = 49,889)** | 1.31 (1.03-1.67) | 0.03 |
| **Postmenopausal women**  **(n = 127,577)** | 1.02 (0.93-1.12) | 0.67 |
| **MHT users**  **(n = 9,183)** | 1.44 (1.00-2.08) | 0.05 |
| **Non-MHT users**  **(n = 118,394)** | 1.00 (0.91-1.10) | 0.99 |
| **Men**  **(n = 186,221)** | 1.00 (0.92-1.08) | 0.91 |
| **<50 years**  **(n = 45,958)** | 0.89 (0.70-1.14) | 0.36 |
| **≥50 years**  **(n = 140,263)** | 1.01 (0.93-1.10) | 0.84 |

Supplemental Table 8 presents Cox proportional hazards models, adjusted for age, age^2^, body mass index, ethnic background, diabetes mellitus, smoking status, cholesterol-lowering medication use, and aspirin use. In addition, models for premenopausal women were further adjusted for OCP use, and models for postmenopausal women were further adjusted for MHT use. aHR, adjusted hazard ratio; CI, confidence interval; Lp(a), lipoprotein(a); MHT, menopausal hormone therapy; OCP, oral contraceptive; VTE, venous thromboembolism

## Supplemental Table 9: Associations of Lp(a) ≥125 nmol/L with incident VTE, accounting for prevalent and incident atrial fibrillation or flutter.

|  | **Incident VTE** | |
| --- | --- | --- |
|  | **aHR (95% CI)** | **P-value** |
| **Premenopausal women**  **(n = 55,173)** | 1.29 (1.02-1.63) | 0.04 |
| **OCP users**  **(n = 4,923)** | 1.52 (0.62-3.68) | 0.36 |
| **Non-OCP users**  **(n = 50,250)** | 1.27 (1.00-1.62) | 0.05 |
| **Postmenopausal women**  **(n = 127,936)** | 1.03 (0.94-1.13) | 0.54 |
| **MHT users**  **(n = 9,227)** | 1.47 (1.02-2.11) | 0.04 |
| **Non-MHT users**  **(n = 118,709)** | 1.01 (0.92-1.11) | 0.88 |
| **Men**  **(n = 185,639)** | 1.01 (0.93-1.09) | 0.90 |
| **<50 years**  **(n = 46,324)** | 0.93 (0.73-1.19) | 0.57 |
| **≥50 years**  **(n = 139,315)** | 1.02 (0.93-1.11) | 0.73 |

Supplemental Table 9 presents Cox proportional hazards models, adjusted for age, age^2^, body mass index, ethnic background, diabetes mellitus, smoking status, cholesterol-lowering medication use, and aspirin use. In addition, models for premenopausal women were further adjusted for OCP use, and models for postmenopausal women were further adjusted for MHT use. All models were adjusted for incident atrial fibrillation or flutter as a time-varying covariate. Participants with prevalent atrial fibrillation or flutter were excluded. aHR, adjusted hazard ratio; CI, confidence interval; Lp(a), lipoprotein(a); MHT, menopausal hormone therapy; OCP, oral contraceptive; VTE, venous thromboembolism

## Supplemental Table 10: Associations of Lp(a) ≥125 nmol/L with incident venous thromboembolism, pulmonary embolism (with or without deep vein thrombosis), deep vein thrombosis (with or without pulmonary embolism), and isolated deep vein thrombosis.

|  | **Incident venous thromboembolism**  **(n = 8,186)** | | **Incident pulmonary embolism (with or without deep vein thrombosis)**  **(n = 5,810)** | | **Incident deep vein thrombosis (with or without pulmonary embolism)**  **(n = 3,366)** | | **Incident isolated deep vein thrombosis**  **(n = 2,376)** | |
| --- | --- | --- | --- | --- | --- | --- | --- | --- |
|  | **aHR (95% CI)** | **P-value** | **aHR (95% CI)** | **P-value** | **aHR (95% CI)** | **P-value** | **aHR (95% CI)** | **P-value** |
| **Pre-menopausal women** | 1.32 (1.04-1.66) | 0.02 | 1.37 (1.04-1.80) | 0.03 | 1.35 (0.94-1.94) | 0.11 | 1.20 (0.77-1.87) | 0.43 |
| **Post-menopausal women** | 1.03 (0.94-1.13) | 0.47 | 1.04 (0.93-1.15) | 0.51 | 0.94 (0.81-1.09) | 0.39 | 1.03 (0.86-1.22) | 0.76 |
| **Men** | 1.00 (0.92-1.08) | 0.94 | 0.98 (0.89-1.08) | 0.68 | 0.97 (0.85-1.09) | 0.58 | 1.01 (0.97-1.05) | 0.60 |

Supplemental Table 10 presents Cox proportional hazards models, adjusted for age, age^2^, body mass index, ethnic background, diabetes mellitus, smoking status, cholesterol-lowering medication use, and aspirin use. In addition, models for premenopausal women were further adjusted for OCP use, and models for postmenopausal women were further adjusted for MHT use. Event counts for each phenotype in the full cohort are reported under the corresponding column. Each phenotype was compared to a reference group of individuals without VTE. aHR, adjusted hazard ratio; CI, confidence interval; Lp(a), lipoprotein(a); MHT, menopausal hormone therapy; OCP, oral contraceptive; VTE, venous thromboembolism

## Supplemental Table 11: Associations of the *LPA* GRS with incident VTE.

|  | ***LPA* GRS (Z-standardized)** | | | | | |
| --- | --- | --- | --- | --- | --- | --- |
|  | **Top 25% vs. remaining 75%** | | **Top 25% vs. bottom 25%** | | **Continuous** | |
|  | **aHR (95% CI)** | **P-value** | **aHR (95% CI)** | **P-value** | **aHR (95% CI)** | **P-value** |
| **Premenopausal women**  **(n = 54,673)** | 1.23 (1.00-1.52) | 0.045 | 1.44 (1.09-1.89) | 0.009 | 1.09 (0.99-1.19) | 0.07 |
| **OCP users**  **(n = 4,876)** | 1.38 (0.66-2.89) | 0.40 | 1.02 (0.41-2.54) | 0.97 | 0.97 (0.67-1.40) | 0.86 |
| **Non-OCP users**  **(n = 49,797)** | 1.22 (0.98-1.51) | 0.07 | 1.48 (1.11-1.98) | 0.007 | 1.09 (1.00-1.20) | 0.05 |
| **Postmenopausal women**  **(n = 127,510)** | 0.99 (0.92-1.08) | 0.90 | 0.95 (0.86-1.05) | 0.35 | 0.99 (0.96-1.03) | 0.59 |
| **MHT users**  **(n = 9,175)** | 1.40 (1.03-1.92) | 0.03 | 2.05 (1.31-3.20) | 0.002 | 1.16 (1.02-1.32) | 0.03 |
| **Non-MHT users**  **(n = 118,335)** | 0.97 (0.89-1.06) | 0.50 | 0.91 (0.82-1.01) | 0.08 | 0.98 (0.94-1.02) | 0.26 |
| **Men**  **(n = 187,063)** | 0.98 (0.91-1.04) | 0.49 | 0.94 (0.87-1.02) | 0.14 | 0.98 (0.96-1.01) | 0.27 |

| **<50 years**  **(n = 46,138)** | 1.17 (0.98-1.41) | 0.09 | 1.18 (0.94-1.49) | 0.15 | 1.07 (0.98-1.15) | 0.11 |
| --- | --- | --- | --- | --- | --- | --- |
| **≥50 years**  **(n = 140,925)** | 0.95 (0.89-1.02) | 0.17 | 0.91 (0.84-0.99) | 0.03 | 0.97 (0.94-1.00) | 0.08 |

Supplemental Table 11 presents Cox proportional hazards models in which the exposure was either Z-standardized *LPA* GRS continuous or the top quartile of *LPA* GRS. Models were adjusted for accounted for age, age^2^, genotyping array, and the first 10 principal components of genetic ancestry. aHR, adjusted hazard ratio; CI, confidence interval; GRS, genetic risk score; Lp(a), lipoprotein(a); MHT, menopausal hormone therapy; OCP, oral contraceptive; VTE, venous thromboembolism

## Supplemental Table 12: Interaction terms for Lp(a) ≥125 nmol/L with log_2_-transformed oestradiol.

|  | **Incident VTE** | |
| --- | --- | --- |
|  | **aHR (95% CI)** | **P-value** |
| **Premenopausal women** | 1.46 (1.21-1.75) | <0.001 |
| **Postmenopausal women** | 1.32 (1.11-1.58) | 0.002 |

Supplemental Table 12 presents Cox proportional hazards models, adjusted for age, age^2^, body mass index, ethnic background, diabetes mellitus, smoking status, cholesterol-lowering medication use, and aspirin use. In addition, models for premenopausal women were further adjusted for OCP use, and models for postmenopausal women were further adjusted for MHT use. aHR, adjusted hazard ratio; CI, confidence interval; Lp(a), lipoprotein(a); VTE, venous thromboembolism

## Supplemental Table 13: Interaction terms for Lp(a) ≥125 nmol/L with log_2_-transformed oestradiol, excluding OCP and MHT users.

|  | **Incident VTE** | |
| --- | --- | --- |
|  | **aHR (95% CI)** | **P-value** |
| **Premenopausal women** | 1.43 (1.18-1.74) | <0.001 |
| **Postmenopausal women** | 1.39 (1.09-1.77) | 0.007 |

Supplemental Table 13 presents Cox proportional hazards models, adjusted for age, age^2^, body mass index, ethnic background, diabetes mellitus, smoking status, cholesterol-lowering medication use, and aspirin use. Premenopausal OCP users and postmenopausal MHT users were excluded. aHR, adjusted hazard ratio; CI, confidence interval; Lp(a), lipoprotein(a); VTE, venous thromboembolism

## Supplemental Table 14: Associations of Lp(a) ≥125 nmol/L with incident VTE across strata of circulating oestradiol levels, excluding OCP and MHT users.

|  | **Incident VTE** | | | |
| --- | --- | --- | --- | --- |
|  | **Unadjusted models** | | **Adjusted models** | |
|  | **HR (95% CI)** | **P-value** | **HR (95% CI)** | **P-value** |
| **Premenopausal women** | | | | |
| **Oestradiol <400 pmol/L**  **(n = 27,057)** | 1.08 (0.77-1.50) | 0.67 | 0.95 (0.68-1.32) | 0.75 |
| **Oestradiol 400-<800 pmol/L**  **(n = 12,590)** | 2.12 (1.31-3.45) | 0.002 | 1.85 (1.13-3.01) | 0.01 |
| **Oestradiol ≥800 pmol/L**  **(n = 6,628)** | 3.00 (1.46-6.16) | 0.003 | 2.90 (1.41-5.97) | 0.004 |
| **Postmenopausal women** | | | | |
| **Oestradiol <175 pmol/L  (n = 106,504)** | 1.03 (0.93-1.14) | 0.57 | 1.00 (0.91-1.11) | 0.93 |
| **Oestradiol ≥175 pmol/L**  **(n = 3,707)** | 1.70 (1.08-2.66) | 0.02 | 1.62 (1.03-2.54) | 0.04 |

Supplemental Table 14 presents Cox proportional hazards models in which participants with Lp(a) <125 nmol/L constitute the reference group. The adjusted models accounted for age, age^2^, body mass index, ethnic background, diabetes mellitus, smoking status, cholesterol-lowering medication use, and aspirin use. Premenopausal OCP users and postmenopausal MHT users were excluded. CI, confidence interval; HR, hazard ratio; Lp(a), lipoprotein(a); MHT, menopausal hormone therapy; OCP, oral contraceptive; VTE, venous thromboembolism
